# Hepatoprotection of *Paederia scandens* (Lour.) Merr. on Acetaminophen-Related Hepatic Injury Rats by ^1^H-NMR-Based Metabonomics Coupled with Network Pharmacology

**DOI:** 10.1155/2022/1375864

**Published:** 2022-08-22

**Authors:** Chao-Ling Tang, Ning Ma, Wan-Ying Sun, Wei Wang, Li-Peng Zhu, Rui-Qi Wang, Jie-Yan Liu, Xiao-Po Zhang

**Affiliations:** ^1^Department of Pharmacy, The First Affiliated Hospital of Hainan Medical University, Haikou 571000, China; ^2^Reproductive Medicine Center, Women and Children Medical Center of Hainan Province, Haikou 571000, China; ^3^Key Laboratory of Tropical Translational Medicine of Ministry of Education, Hainan Key Laboratory for Research and Development of Tropical Herbs, School of Pharmacy, Hainan Medical University, Haikou 571199, China; ^4^Department of Pharmacy, Yongzhou Central Hospital, Yongzhou 425000, China

## Abstract

**Background:**

Acetaminophen-related hepatic injury (ARHI) is a kind of acute hepatic injury caused by overdosing acetaminophen, which is mainly related to toxic metabolite production, oxidative stress, and mitochondrial dysfunction. The extract of *Paederia scandens* (Lour.) Merr. (PSM) has the abilities of anti-inflammatory, antivirus, and antioxidation. Research studies showed that PSM could improve acute or chronic hepatic injury, while the mechanism of which is still indistinct.

**Methods:**

Here, the authors applied the approach based on serum metabonomics combined with network pharmacology to study the protection of PSM on ARHI rats.

**Results:**

10 serum potential biomarkers were found to be closely related to ARHI by metabonomics, while 3 compounds (L-ascorbyl 2,6-dipalmitate, squalene, and tributyl O-acetylcitrate) and 3 targets (NOS2, MAOB, and PDE3A) were found that might be the potential active components and active site of PSM on treating ARHI by network pharmacology analysis. Furthermore, molecular biology strategy was performed to validate whether iNOS/NF-*κ*B signaling pathway is the potential mechanism of PSM treating ARHI.

**Conclusions:**

This study indicated that PSM could ameliorate ARHI by iNOS/NF-*κ*B signaling pathway. During ARHI treatment by PSM, L-ascorbyl 2, 6-dipalmitate, squalene, and tributyl O-acetylcitrate might be the potential active components, while the possible active site might be NOS2, MAOB, and PDE3A.

## 1. Introduction

*Paederia scandens* (Lour.) Merr. (PSM), a traditional Chinese medicine also used as a vegetable, is a member of the “generally recognized as safe” (GRAS) category of plants used for therapeutic purposes [1, 2]. The tender stems and leaves of PSM and Hainanese “chicken excrement fragrant cake” made with PSM are consumed as local traditional food. PSM has both edible and medicinal values. The root, leaf, stem, and fruit extracts are commonly used to treat liver disease, jaundice, dysentery, and abdominal mass [3–5]. Several studies have shown that PSM and its extracts exert antioxidant and hepatoprotective effects via downregulating heat shock cognate 71 kDa protein (hsp7°C) [6]. PSM, described as a “medicine and food homology” species, may serve as a potential natural resource for developing effective hepatoprotective treatments and additives for functional health food. However, at present, little is known about the specific mechanisms of action of PSM. In this study, an acetaminophen-induced acute hepatic injury rat model was generated for elucidating the potential therapeutic effects of PSM and its underlying mechanisms.

Acetaminophen-related liver injury (ARHI) is a typical representation of drug-induced liver injury. Animal models provide an important tool for exploring the pathways of drug-induced liver injury and therapeutic activities of hepatoprotective drugs [7]. Over the last 50 or so years, several researchers have focused on the mechanisms underlying hepatotoxicity of ARHI [7, 8]. Here, we introduced a high-throughput and rapid metabonomics approach to clarify the pathways of hepatotoxicity of acetaminophen and protective effects of PSM. We aimed to use network pharmacology based on bioinformatics analysis to screen and identify the potential targets of PSM for preliminary validation of its hepatoprotective mechanisms.

Metabonomics is a rapidly developing field of systems biology that mainly analyzes the response of endogenous metabolites to external stimuli based on global metabolite profiles in biologic samples, such as urine, plasma, or tissue [9]. The holistic and comprehensive features of metabonomics reflect metabolic profiles within biologic systems and focus on potential biomarkers and metabolic pathways to establish the pathways associated with pathology, facilitating understanding of the complex mechanisms underlying the therapeutic effects of traditional Chinese medicine [10, 11]. ^1^H-NMR spectroscopy provides a high-throughput technique for analysis of biofluids and tissues. Thus, metabonomics based on ^1^H-NMR presents an effective approach for screening potential biomarkers and targets of PSM on ARHI. Furthermore, network pharmacology is a powerful tool for elucidating the complex and holistic mechanisms of action of herbal medicines that can provide information on the multitarget therapeutic pathways of TCM through (1) constructing disease-specific networks at the molecular level based on published and high-throughput omics data and (2) combining active compounds at the system level to predict therapeutic targets, followed by clarification of the active compounds and therapeutic mechanisms of TCM from a holistic viewpoint [12, 13]. Therefore, combining experimental evidence obtained using metabonomics analysis with systematic network pharmacology predictions could aid in understanding the therapeutic effects of PSM against ARHI and the underlying mechanisms.

In this study, a ^1^H-NMR-based metabonomics-coupled network pharmacology approach was applied to investigate the mechanisms of action of PSM on ARHI using a rat model. Metabolic pathway and comprehensive metabolic network analyzes revealed relevant pathways and provided a novel breakthrough in terms of identification of potential therapeutic targets. Additionally, the PSM-ARHI-metabolic pathway-target network was constructed via network pharmacology to visualize the potential therapeutic targets of PSM. To our knowledge, this is the first study to utilize metabonomics coupled with network pharmacology for elucidating the hepatoprotective mechanisms of PSM activity against ARHI.

## 2. Materials and Methods

### 2.1. Materials

*Paederia scandens* (Lour) Merr. (place of origin: Hubei, batch no. 181201) was purchased from Hainan Linshishengtai Pharmaceutical Co. Ltd. Acetaminophen tablets (batch no. 190402) were acquired from Sinopharm Group Guangdong Medi-World Pharmaceutical Co., Ltd. Silybin capsules (batch no. 850703059) were purchased from Tianjin Tasly Sants Pharmaceutical Co., Ltd. AST and ALT assay kits were obtained from Nanjing Jiancheng Bioengineering Institute. Rat ELISA kits of iNOS (LOT202005), IL-10 (LOT202006), IL-6 (LOT202006), TNF-*α* (LOT202006), and NF-*κ*B (LOT202006) were obtained from Shanghai Enzyme-Linked Biotechnology Co., Ltd. Male Sprague Dawley rats were purchased from Changsha Tianqin Biological Technology Co., Ltd. Our study was performed according to the international, national, and institutional guidelines for animal experiments. The animal protocol was approved by the Ethics Review Committee for Animal Experimentation of Hainan Medical University. The ethical inspection number is HYLL-2021-387.

### 2.2. Extract Preparation

Dried herbs of PSM were added to water (w/v, 1 : 10) for 30 min of soaking followed by 60 min of boiling. After extraction, filters were used to prepare a concentration of 0.23 g/mL PSM extract for animal experiments. Component characterization of PSM is described in Supplementary material (Figure S1).

### 2.3. Animal Experiments

A total of 30 male SD rats (180–220 g) were randomly divided into 5 groups. Rats in the control and model groups were administered distilled water (1 mL/100 g, i.g), while the PSM-L, PSM-H, and positive drug groups were administered 0.7 g/kg PSM, 2.3 g/kg PSM, and 30 mg/kg silymarin daily (1 mL/100 g, i.g), respectively. After 7 days of successive administration, all rats except those in the control group were administered 2000 mg/kg acetaminophen (1 mL/100 g, i.g) to replicate the acetaminophen-induced acute hepatic injury model.

### 2.4. Sample Collection and Biochemical Parameters

At 24 h after acetaminophen administration, blood samples were obtained from abdominal aorta and serum samples were prepared by centrifugation for 10 min at 4°C for 3,500 rpm. The ALT and AST contents were determined using specific assay kits. The serum iNOS, IL-10, IL-6, TNF-*α,* and NF-*κ*B were measured by ELISA kits. The remaining serum was stored at −80°C for further analysis. Liver tissues were washed with cold normal saline and stored at −80°C for further use.

### 2.5. Histopathology of Liver

Sections of liver were fixed in 10% formaldehyde and embedded in paraffin wax. After HE staining, pathological changes in liver tissues were observed via microscopy. Images of pathological sections were obtained at x100 magnification.

### 2.6. Sample Preparation

Serum samples (300 *μ*L) from the PSM-H group and 300 *μ*L PBS (pH 7.4, containing 1 : 1 double-distilled water and D_2_O) were mixed and vortexed. The mixture was centrifuged for 10 mins at 4°C for 10,000 rpm, and the supernatant was isolated and collected in 5 mm NMR tubes. Prepared samples were stored at 4°C until ^1^H-NMR analysis.

### 2.7. Test Conditions for ^1^H-NMR

The test conditions for ^1^H-NMR are presented in Table 1.

### 2.8. Data Processing and Pattern Recognition

Baseline correction and phase adjustment for all spectrograms were performed using MestReNova software (Mestrelab Research, Santiago de Compostela, Spain). The chemical shift was calibrated with lactic acid low field peak (*δ* = 1.336 ppm) and peak valley (*δ* = 1.330 ppm). The water peak (*δ* = 4.55–5.15 ppm) was removed, and spectra were segmented at 0.04 ppm intervals across the chemical shift (*δ*) of 0.04–9.00 ppm. Raw data were exported as a .txt format after normalization and imported into SIMCA-P 14.1 for multivariate analysis. The *t*-test was used to evaluate significant differences in potential biomarkers.

### 2.9. Identification of Potential Biomarkers and Construction of a Metabolic Network

Identification and characterization of potential biomarkers were achieved by comparing H–H COSY, HSQC, and the standard spectrograms from the HMDB database (https://hmdb.ca/). A comprehensive metabolic network was constructed by integration of potential biomarkers using KEGG (https://www.genome.ad.jp/kegg/) and MetaboAnalyst 4.0 (https://www.metaboanalyst.ca/).

### 2.10. Network Pharmacology Analysis

The chemical components of PSM were screened (oral bioavailability ≥30% and drug likeness, DL ≥ 0.18) using the TCMSP database (https://tcmspw.com/tcmsp.php). The SMILE format of compounds was imported into SwissTargetPrediction database (https://www.swisstargetprediction.ch/) for target prediction, and a compound-target table was obtained to generate the PSM-component-target network.

The GeneCards database (https://www.genecards.org/) was selected for screening the relevant target genes of ARHI. The table of relevant target genes was subsequently exported. Scores >10 were used to construct the disease-target network.

Genes of the relevant enzymes involved in key metabolic pathways were searched using the KEGG database based on the results of metabonomics and an ARHI-relevant metabolic pathway-target network constructed.

The PSM-component-disease-metabolic pathway-target network was conducted by merging the PSM-component-target, disease-target, and metabolic pathway-target networks.

### 2.11. Molecular Docking on Compounds and Targets of PSM

To screen for potential active components of PSM, molecular docking technology was performed to mimic binding patterns and compare the binding scores between proteins and ligands. The common targets of PSM and metabonomics and the targets among PSM, hepatic injury, and metabonomics were selected as proteins for docking and screened PSM components (same as network pharmacology analysis) taken as the ligands.

### 2.12. Statistical Analysis

Statistical analysis was performed using SPSS 20.0 software. The results were evaluated as mean ± standard deviation and significant differences among groups determined using one-way analysis of variance (ANOVA). The threshold value of differences was set as *P* < 0.05.

## 3. Results

### 3.1. Effects of PSM on Hepatic Biochemical Parameters

Following acetaminophen administration, the liver of rats is adversely affected due to N-acetyl-p-benzoquinone imine (NAPQI) metabolite-induced damage. Aspartate aminotransferase (AST) and alanine aminotransferase (ALT) levels in rat serum were detected to assess the injury situation of liver. Our results (Table 2) showed that compared with the control group, AST and ALT levels were significantly elevated in the model group and downregulated in the positive drug group. In the PSM-L and PSM-H groups, AST and ALT levels were significantly decreased, particularly in the PSM-H group relative to the PSM-L and positive drug groups.

### 3.2. Effects of PSM on Hepatic Histopathology

**Figure 1:**
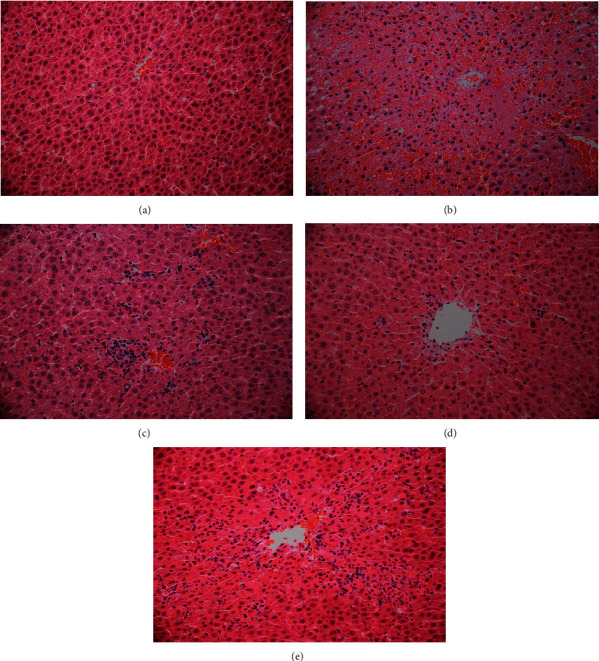
Hepatic histopathology of rats in different groups (x100). (a) Control group, (b) model group, (c) PSM-H group, (d) PSM-L group, and (e) silymarin group.

Based on the histopathology data, liver tissue of rats in the control group displayed normal and clear lobular architecture with cord-shaped hepatic cells arranged radially around the central veins, no degeneration, necrosis, or congestion of hepatocytes, and no infiltration of inflammatory cells (Figure 1(a)). Series of damage such as large-scale necrosis around the central hepatic lobule vein, pyknosis or dissolution of the nucleus, destruction of hepatocyte cord morphology, infiltration of inflammatory cells, and significant congestion was observed in the model group after treatment with acetaminophen (Figure 1(b)). Notably, infiltration of inflammatory cells, congestion, and spotty necrosis of hepatocytes were evident in terms of hepatic histopathology of PSM-treated rats, indicating that PSM induced a significant improvement of ARHI (Figures 1(c) and 1(d)). Similar protective effects were observed in silymarin-treated rats (Figure 1(e)).

### 3.3. Characterization of ^1^H-NMR Spectra

**Figure 2:**
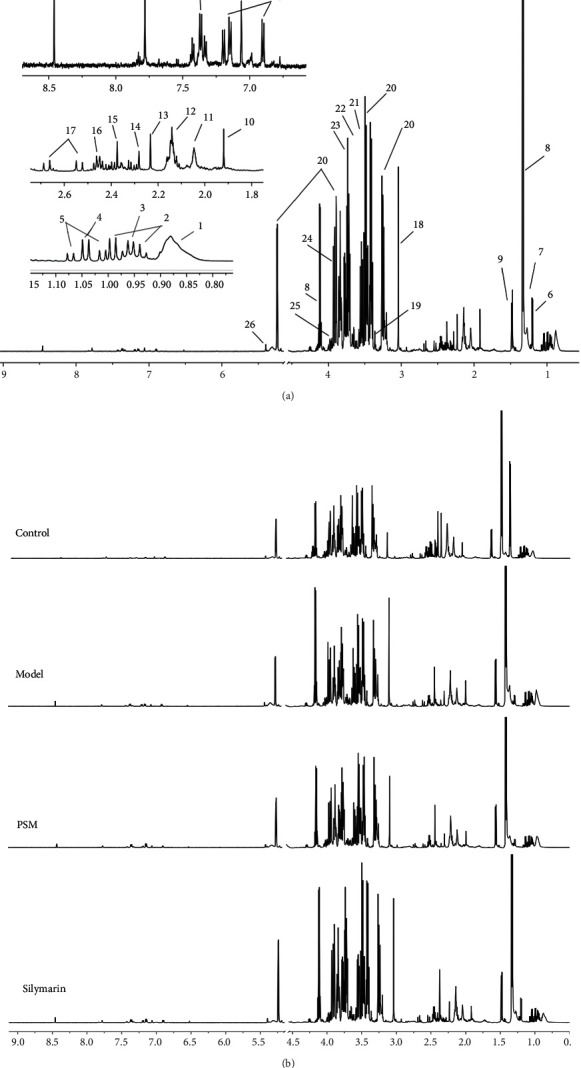
Characterization of 1H-NMR spectra (a) and representative 1H-NMR spectra of different groups (b). (1. lipid; 2. isoleucine; 3. leucine; 4. isobutyrate; 5. valine; 6. 3-hydroxybutyrate; 7. methylmalonate; 8. lactate; 9. alanine; 10. acetate; 11. N-acetyl groups; 12. O-acetyl groups; 13. acetone; 14. acetoacetate; 15. pyruvate; 16. glutamine; 17. citrate; 18. creatine; 19. methanol; 20. glucose; 21. glycerol; 22. threonine; 23. guanidoacetic acid; 24. glycolate; 25. aspartic acid; 26. glycogen; 27. tyrosine; 28. histidine; 29. phenylalanine; 30. formic acid).

^1^H-NMR analysis was performed on serum samples of control, model, and PSM-treated groups representing the normal and pathological states and protective effects, respectively. Characterization of ^1^H-NMR spectra and representative ^1^H-NMR spectra is shown in Figure 2, respectively. In total, 30 metabolites were identified: 1. lipid; 2. isoleucine; 3. leucine; 4. isobutyrate; 5. valine; 6. 3-hydroxybutyrate; 7. methylmalonate; 8. lactate; 9. alanine; 10. acetate; 11. N-acetyl groups; 12. O-acetyl groups; 13. acetone; 14. acetoacetate; 15. pyruvate; 16. glutamine; 17. citrate; 18. creatine; 19. methanol; 20. glucose; 21. glycerol; 22. threonine; 23. guanidoacetic acid; 24. glycolate; 25. aspartic acid; 26. glycogen; 27. tyrosine; 28. histidine; 29. phenylalanine; and 30. formic acid.

### 3.4. Multivariate Statistical Analysis

**Figure 3:**
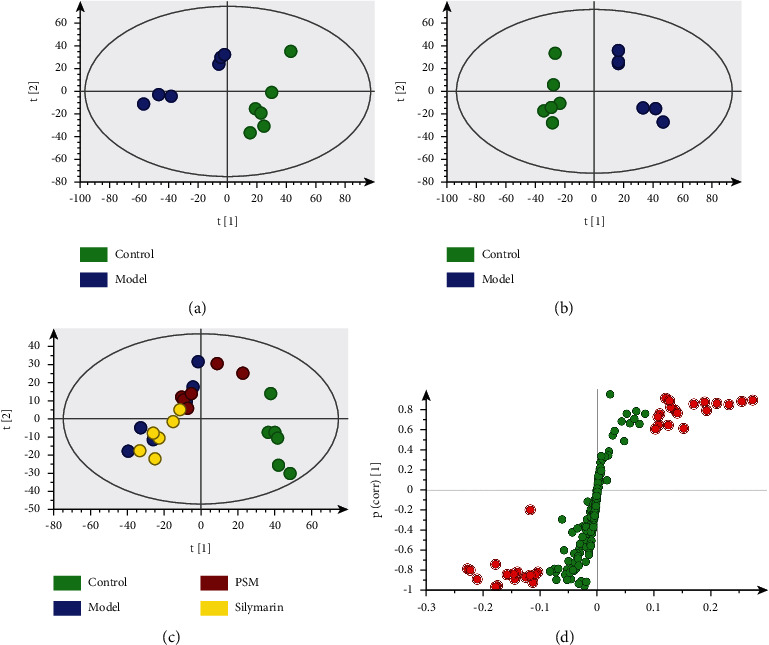
Multivariate statistical analysis of metabonomics. ((a) PCA score plot of control and model group, (b) PLS-DA score plot of control, model, PSM, and silymarin groups, (c) PLS-DA score plot of control group and model group, (d) S-plot) (control: control group, model: model group, PSM : PSM group, silymarin: silymarin group).

Raw data were exported from MestReNova software and mean-centered and Pareto-scaled for multivariate analysis. Principal component analysis (PCA) was conducted to determine whether two groups could be separated, and partial least squares-discriminate analysis (PLS-DA) was performed to magnify metabolic patterns and differences between two groups. The PCA score plot (Figure 3(a)) showed complete separation between the control and model groups, indicative of distinct metabolic profiles. According to the PLS-DA score plots of the control, model, PSM, and silymarin groups (Figure 3(b)), metabolic profiles of the model and control groups were different, indicating that acetaminophen-triggered injury influences the biochemical changes in rats. However, the metabolic profiles of the PSM and control groups were comparable, suggesting that PSM exerts beneficial effects through modulating the disordered metabolome. To validate the differences and identify metabolites potentially related to ARHI, PLS-DA was conducted for the control and model groups (Figure 3(c)). The chemical shift in potential metabolites was screened under conditions of variable importance of project (VIP) > 1 and *p* of S-plot >|0.1| (Figure 3(d)). To prevent against model overfitting, the performance of the PLS-DA models between the control group and the model group was evaluated using sevenfold cross-validation and 200 times permutation (Figure S2). Values of the parameters for evaluating modeling quality are listed in Table 3. The results suggested that models established in this study had good differentiating, fitness, and prediction.

Overall, 10 metabolites were identified as potential biomarkers. By comparison with the HMDB database, published evidence, and ^1^H–^1^H COSY (Figure S3), the 10 biomarkers were identified as isoleucine, 3-hydroxybutyric acid, methylmalonic acid, lipid, acetone, acetoacetate, pyruvic acid, glutamine, aspartic acid, and glucose (Table 4). In the serum of ARHI rats, isoleucine, lipid, aspartic acid, and glucose levels were elevated, while 3-hydroxybutyric acid, methylmalonic acid, acetone, acetoacetate, pyruvic acid, and glutamine were reduced. PSM treatment induced a significant decrease in the lipid and glucose levels.

### 3.5. Metabolic Pathway Analysis

**Figure 4:**
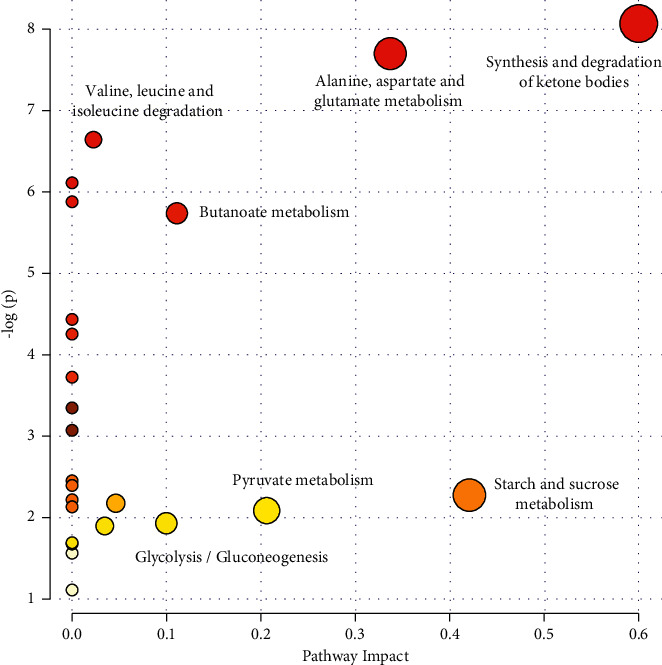
Importance of ARHI-related metabolic pathways.

**Figure 5:**
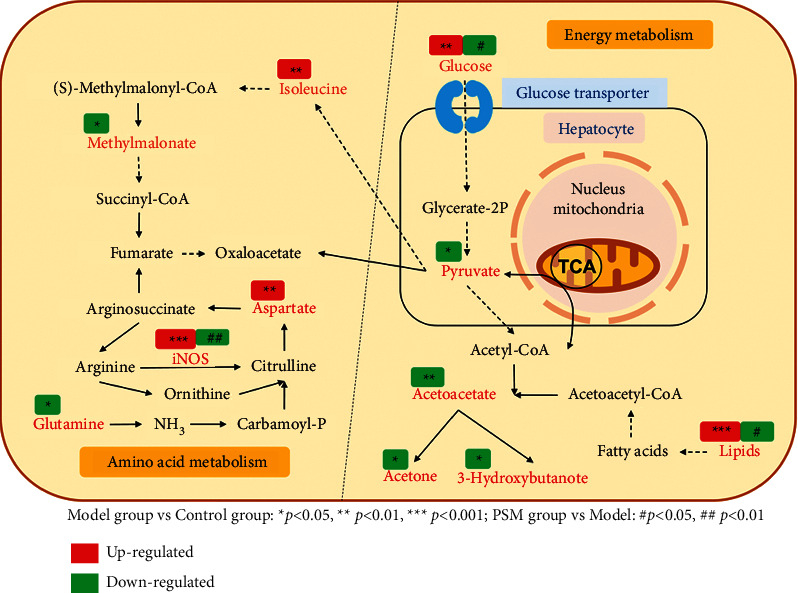
Comprehensive metabolic pathway network of ARHI.

To elucidate the associations between potential biomarkers and metabolic pathways, we integrated all identified biomarkers to conduct metabolic pathway analysis using MetaboAnalyst 4.0. Six metabolic pathways, including synthesis and degradation of ketone bodies, starch and sucrose metabolism, alanine, aspartate and glutamate metabolism, pyruvate metabolism, butanoate metabolism, and glycolysis/gluconeogenesis, were associated with ARHI (Figure 4). We further constructed a comprehensive metabolic pathway network of ARHI (Figure 5), with a view to clarifying the regulatory effects of PSM on ARHI.

### 3.6. Construction of PSM-Component-Disease-Pathway-Target Networks

**Figure 6:**
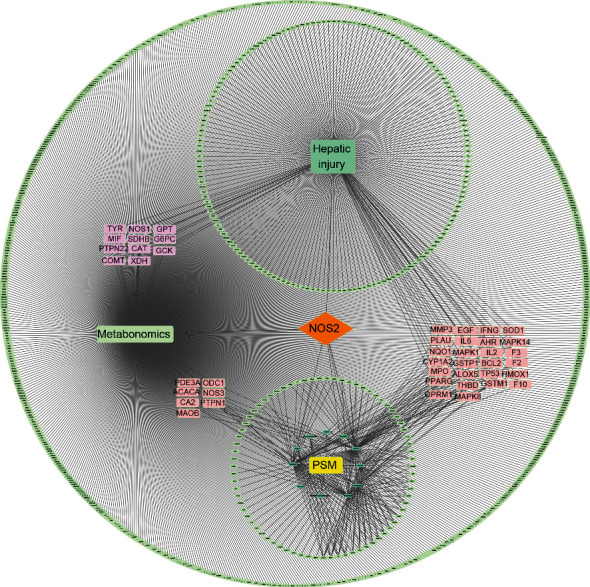
PSM-component-disease-metabolic pathway-target network.

By merging the PSM-component-target, disease-target, and metabolic pathway-target networks, the PSM-component-disease-pathway-target network was generated (Figure 6). Notably, NOS2 was the only common target among the PSM, ARHI, and metabolic pathways. Seven targets, including PDE3A, ACACA, MAOB, CA2, ODC1, NOS3, and PTPN1, were intersectional targets of PSM and metabolic pathways. Furthermore, 11 mutual targets (TYR, MIF, PTPN22, COMT, NOS1, SDHB, CAT, XDH, GPT, G6PC, and GCK) of ARHI and metabolic pathways and 26 (AHR, MAPK1, TP53, SOD1, BCL2, PLAU, HMOX1, OPRM1, MMP3, IFNG, PPARG, EGF, F3, IL-6, GSTP1, F2, IL-2, CYP1A2, MPO, GSTM1, NQO1, MAPK14, MAPK8, F10, THBD, and ALOX5) common targets of PSM and ARHI were identified.

### 3.7. Discovery of Potential Active Components and Targets of PSM via Molecular Docking Technology

**Figure 7:**
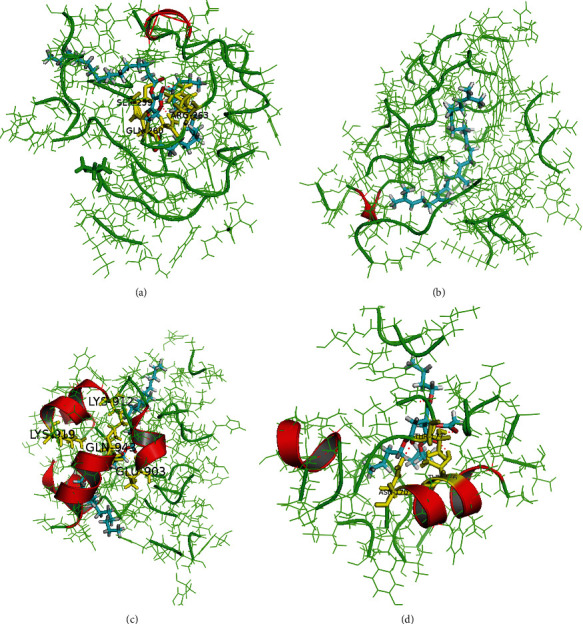
Molecular docking sites of potential active compounds and targets ((a) L-ascorbyl 2,6-dipalmitate binding with NOS2, (b) squalene binding with NOS2, (c) L-ascorbyl 2,6-dipalmitate binding with PDE3A, and (d) tributyl O-acetylcitrate binding with MAOB).

While the protective mechanisms of action of PSM against ARHI in our rat model were initially investigated using network pharmacology analysis, available information on the active components of PSM is still limited. Components with binding scores >10 were selected as potential active compounds for ARHI. Three compounds were characterized as the potential active components (L-ascorbyl 2,6-dipalmitate, squalene, and tributyl O-acetylcitrate) and three proteins as active targets (NOS2, MAOB, and PDE3A) of PSM involved in protective activity against ARHI (Figure 7).

### 3.8. The Protective Effect of PSM Is Potentially Exerted through iNOS/NF-*κ*B Signaling

**Figure 8:**
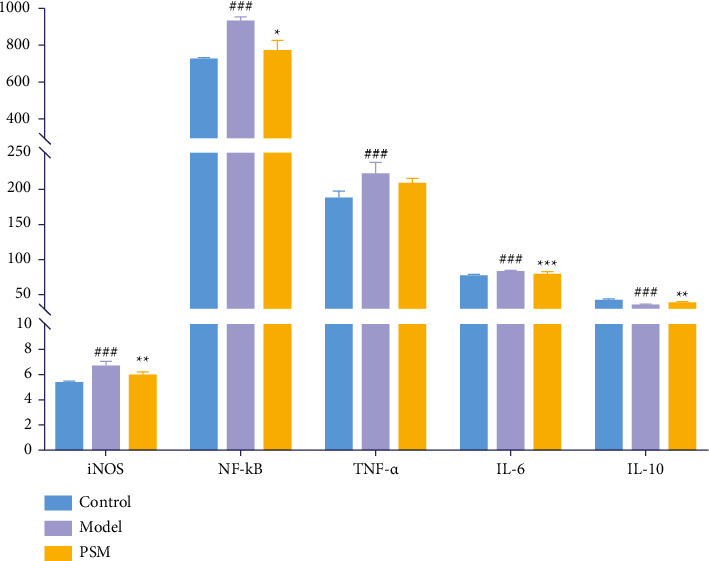
Content histogram of iNOS, NF-*κ*B, TNF-*α*, IL-6, and IL-10 in rats' serum. (Compared to control group as follows: ^#^*p* < 0.05, ^##^*p* < 0.01, ^###^*p* < 0.001, compared to model group as follows: *∗p* < 0.05, ^*∗∗*^*p* < 0.01, ^*∗∗∗*^*p* < 0.001).

As NOS2 was the only common target identified among PSM, ARHI, and metabolic pathways, we examined the content of iNOS encoded by NOS2 in rats. iNOS, IL-6, and TNF-*α* are activators of NF-*κ*B, which mediates the inflammatory response. IL-10 acts as an anti-inflammatory cytokine through inhibiting the release of inflammatory mediators by mononuclear macrophages and promoting the release of anti-inflammatory factors. Accordingly, the IL-6, NF-*κ*B, and IL-10 contents in rat serum were determined to clarify the mechanisms of action of PSM against ARHI. Our collective results suggest that PSM protects against ARHI through modulating the iNOS/NF-*κ*B signaling pathway, specifically inducing a decrease in iNOS, inflammatory factors, and NF-*κ*B and elevation of anti-inflammatory cytokines, such as IL-10 (Figure 8).

## 4. Discussion

In this study, ^1^H-NMR-based metabonomics was combined with the network pharmacology strategy to explore the hepatoprotective effect and potential mechanisms of action of PSM in ARHI rats. Administration of an overdose of oral acetaminophen led to the destruction of hepatocytes and normal hepatocyte cords in rat liver tissue, the infiltration of inflammatory cells, and the significant increase in AST and ALT in serum, strongly supporting the induction of liver injury in animals. Notably, after treatment with PSM, inflammatory infiltration of liver tissue was alleviated, cell necrosis was reduced, and AST and ALT levels in blood were reduced, indicating that PSM improves acute liver injury induced by acetaminophen.

To elucidate the mechanisms by which PSM improves ARHI, ^1^H-NMR-based metabonomics research combined with multivariate statistical chemometrics was applied to characterize the serum metabolic profile of rats in each group. The metabolic profiles of the blank, model, and PSM groups were examined, and ten potential biomarkers closely related to ARHI were identified as 3-hydroxybutyric acid, methylmalonic acid, lipid, acetone, acetoacetate, pyruvic acid, glutamine, aspartic acid, and glucose. The data suggest disruption of energy and amino acid metabolism in ARHI rats. PSM significantly regulated the lipid and glucose contents, indicating that its therapeutic effects are related to the modulation of abnormal metabolic levels in rats.

According to previous studies, the hepatotoxicity of high-dose acetaminophen is mainly attributed to the generation of the toxic metabolite N-acetyl-p-benzoquinone imine (NAPQI) in the liver, which combines with glutathione to exert a detoxification effect. Upon depletion of glutathione, NAPQI continues to bind cellular proteins, in particular, mitochondrial proteins [14, 15], inhibiting beta oxidation of mitochondrial fatty acids [16] that results in massive necrosis and apoptosis of hepatocytes and disruption of fatty acid, glycerophospholipid, and energy metabolism [17]. These results support the relevance of energy metabolism dysfunction in the pathogenesis of ARHI. In our experiments, levels of glucose and lipid in serum of the model group were increased, while pyruvic acid, acetoacetate, acetone, and 3-hydroxybutyric acid were significantly decreased. Combined with the findings of earlier studies, our data indicate that rats treated with acetaminophen experience disorders of glucose and lipid metabolism and mitochondrial dysfunction. Treatment with PSM effectively modulated abnormal glucose and lipid levels triggered by acetaminophen in rats, suggesting that PSM improves ARHI via regulatory effects on abnormal energy metabolism.

Here, we have identified for the first time the potential active compounds (L-ascorbyl 2,6-dipalmitate, squalene, and tributyl O-acetylcitrate) and targets (NOS2, MAOB, and PDE3A) of PSM that contributes to its beneficial effects against ARHI. Among these factors, NOS2 is a widely characterized target in pathogenesis and therapy. Research on the influence of iNOS on ARHI using iNOS-null mice and molecular biology experiments suggests that the hepatotoxicity of acetaminophen is mediated by peroxynitrite and superoxide related to iNOS [18, 19]. The group of Kamanaka examined the protective effects of the iNOS inhibitor, ONO-1714, against ARHI. Their experiments confirmed that NO produced by iNOS plays important roles in the pathogenesis and therapy of ARHI [20]. Accordingly, we speculated that L-ascorbyl 2,6-dipalmitate and squalene are active components of PSM that inhibit iNOS, in turn, contributing to its hepatoprotective effects. *PDE3A* encodes phosphodiesterase, which is widely distributed in hepatocytes, vascular endothelial cells, and platelets, and involved in AMP/GMP production in purine metabolism. An earlier study showed that the PDE3 inhibitor amrinone enhances the physiological effects (including vasodilation, inhibition of free radical generation and inflammatory cytokine production, suppression of neutrophil activation, and prevention of platelet aggregation) of adenosine, potentially through mediating intracellular signals, such as cAMP, cGMP, and NO [21]. These activities may underlie the mechanism of action of the PDE3 inhibitor against ischemia/reperfusion liver injury and its activity as a hepatoprotective agent. Based on our data in conjunction with the results of molecular docking, we hypothesized that L-ascorbyl 2,6-dipalmitate inhibits not only iNOS but also PDE3 to protect against hepatic injury. *MAOB* is the gene encoding monoamine oxidase (MAO). *MAOB* is reported to play a role in the pathogenesis and therapy of depression, and effects are exerted through inhibiting MAO and degradation of 5-HT and suppressing the production of H_2_O_2_. Considering the pathogenesis of ARHI is related to superoxide production, tributyl O-acetylcitrate in PSM could inhibit *MAOB* and suppress superoxide production to exert hepatoprotective activity.

To explore the potential targets of PSM for therapeutic activity against ARHI, network pharmacology analysis technology was applied to screen the common target, NOS2, of PSM, ARHI, and metabonomics pathways. The *NOS2* gene encodes iNOS, an enzyme that mediates the production of NO. Gardner et al. [22] reported that iNOS and NO levels in rat hepatocytes are increased after acetaminophen treatment and hepatotoxicity is counteracted upon pretreatment with an iNOS inhibitor, suggesting a critical role of NO in hepatotoxicity induced by acetaminophen. In addition, excessive use of acetaminophen has been shown to promote transcriptional activation of pro-inflammatory factors (such as TNF-*α* and IL-1*β*) in macrophages. iNOS and pro-inflammatory factors activate NF-*κ*B, which induces inflammatory cytokine production and, subsequently, inflammation finally leading to liver damage [23–26]. In this study, we measured the levels of pro-inflammatory cytokines (IL-6 and TNF-*α*) and anti-inflammatory factors (IL-10, iNOS, and NF-*κ*B) in serum. Serum levels of iNOS, NF-*κ*B, and pro-inflammatory factors were significantly increased, while anti-inflammatory factors were significantly decreased in acetaminophen-treated rats, which were reversed following PSM treatment. These findings suggest that PSM plays a protective role in liver through regulating the iNOS/NF-*κ*B signaling pathway, consistent with our metabonomics and network pharmacology prediction results.

## 5. Conclusion

The therapeutic effect of PSM against ARHI was validated and potential biomarkers and metabolic pathways associated with ARHI were determined in our preliminary study. Our results suggest that the hepatoprotective effect of PSM is closely associated with regulation of iNOS/NF-*κ*B signaling and regulation of abnormal energy and amino acid metabolism. Data from this study provide a scientific basis for the development of PSM as a novel therapeutic drug for ARHI. However, further research is required to gain further insights into the precise molecular mechanisms underlying the hepatoprotective effects of PSM.

## Figures and Tables

**Table 1:** Test condition of ^1^H-NMR.

No.	Parameter	Value	No.	Parameter	Value
1	Title	CPMG	7	Relaxation delay	4s
2	Origin	Bruker AVIII HD	8	Acquisition time	3.0671s
3	Solvent	D2O	9	Spectral width	12019.2 Hz
4	Temperature	298K	10	Number of scans	64
5	Pulse sequence	CPMG	11	Nucleus	1H
6	Experiment	1D			

**Table 2:** Hepatic biochemical parameters among different groups (*n* = 6).

	Control group	Model group	PSM-H group	PSM-L group	Silymarin group
ALT	8.54 ± 2.97	134.72 ± 121.74^###^	35.46 ± 15.72^*∗∗∗*^	33.65 ± 16.50^*∗*^	95.03 ± 66.02^*∗∗*^
AST	50.40 ± 10.50	178.00 ± 79.82^##^	76.55 ± 33.51^*∗*^	110.89 ± 20.86^*∗*^	105.51 ± 46.78

Model group *vs.* control group as follows: ^#^*p* < 0.05,^##^*p* < 0.01,^###^*p* < 0.001. PSM and silymarin group *vs.* model group as follows: *∗p* < 0.05, ^*∗∗*^*p* < 0.01, ^*∗∗∗*^*p* < 0.001.

**Table 3:** Summary of the evaluation of the model quality (A: the number of components).

	PLS-DA			
	A	R2X	R2Y	Q2 (cum)
Control vs model	3	0.855	0.985	0.935
PSM vs model	3	0.784	0.922	0.660
Silymarin vs model	3	0.793	0.894	0.504

**Table 4:** Information of ARHI-related potential biomarkers.

No.	Chemical shift	VIP	Metabolites	Model vs. control	PSM vs. model	Silymarin vs. model	Related pathway
1	0.95/1.02	2.05	Isoleucine	↑^###^	—	↓^*∗*^	Valine, leucine, and isoleucine degradation
2	1.2	3.54	3-Hydroxybutyrate	↓^#^	—	—	Synthesis and degradation of ketone bodies/butanoate metabolism
3	1.24	3.45	Methylmalonate	↓^#^	—	—	Valine, leucine, and isoleucine degradation
4	0.86/1.28/5.28	1.64	Lipid	↑^###^	↓^*∗*^	↓^*∗*^	Lipid metabolism
5	2.24	2.24	Acetone	↓^#^	—	—	Synthesis and degradation of ketone bodies/butanoate metabolism
6	2.28	1.65	Acetoacetate	↓^##^	—	—	Synthesis and degradation of ketone bodies/butanoate metabolism
7	2.32	2.78	Pyruvate	↓^#^	—	—	Pyruvate metabolism/alanine, aspartate, and glutamate metabolism
8	2.44	1.93	Glutamine	↓^#^	—	—	Alanine, aspartate, and glutamate metabolism
9	3.92	2.64	Aspartic acid	↑^##^	—	—	Alanine, aspartate, and glutamate metabolism
10	3.48/3.82/5.24	2.9	Glucose	↑^##^	↓^*∗*^	—	Glycolysis/gluconeogenesis/starch and sucrose metabolism

Compared to control group as follows: ^#^*p* < 0.05,^##^*p* < 0.01,^###^*p* < 0.001; compared to model group as follows: *∗p* < 0.05, ^*∗∗*^*p* < 0.01, ^*∗∗∗*^*p* < 0.001.

## Data Availability

The datasets used and/or analyzed during this study are available from the corresponding author on reasonable request.

## Abbreviations

ANOVA: One-way analysis of variance
ARHI: Acetaminophen-related hepatic injury
MAO: Monoamine oxidase
NAPQI: N-acetyl-p-benzoquinone imine
PCA: Principal component analysis
PLS-DA: Partial least squares-discriminate analysis
PSM: *Paederia scandens* (Lour.) Merr.
TCM: Traditional Chinese medicine.
VIP: Variable importance of project.

## Supplementary Materials

Supplementary Materials
*Figure S1*. TIC of the water extract of PSM and the three identified compounds. *Figure S2.* Cross-validation of the PLS-DA model (using 200 random permutations) to evaluate the validity of the mode. *Figure S3.*^1^H-^1^H COSY and HSQC of 2D ^1^H-NMR.
