# Supplementary material for: Hepatoprotection of Paederia scandens (Lour.) Merr. on Acetaminophen-Related Hepatic Injury Rats by 1H-NMR-Based Metabonomics Coupled with Network Pharmacology
Source: Evid Based Complement Alternat Med. 2022 Aug 22;2022:1375864. doi: 10.1155/2022/1375864 (PMC9423956; doi:10.1155/2022/1375864)
Supplement: Supplementary Materials — Figure S1. TIC of the water extract of PSM and the three identified compounds. Figure S2. Cross-validation of the PLS-DA model (using 200 random permutations) to evaluate the validity of the mode. Figure S3.1H-1H COSY and HSQC of 2D 1H-NMR. [file 1375864.f1.doc]

**Supplemental material**

1. The characterization of chemical profile of *Paederia scandens* (Lour.) Merr. extract by UPLC-QTOF-MS

1.1 Chemicals

Pure water was from a manufacture of ultrapure water system (Chengdu Yue Chun Technology Co., Ltd; Chengdu, China). Formic acid was obtained from ChengDu KeLong Chemical Company (Chengdu, China). Acetonitrile (HPLC grade) was purchased from Thermo Fisher Scientific Inc. (Shanghai, China).

1.2 Extraction of PSM

Dry herbs of PSM were added water (w/v = 1:10) for 30 min soaking and then 60 min boiling. After extracting, the filters were used to prepare a concentration of 0.23g/ml PSM extracts.

1.3 Sample preparation

The water extract of PSM was dissolved in methanol (2.0 mg/mL) for UPLC-QTOF-MS analysis.

1.4 UPLC-QTOF-MS Conditions

The LC-MS analysis was performed on a Waters ACQUITY UPLC I-Class system coupled to a Waters Xevo G2XS QTOF MS system. Chromatographic separation was achieved using a Waters BEH C18 (2.1 mm × 100 mm, 1.7 μm particle) analytical column operated at 40 °C. The mobile phase consisted of 0.1 formic acid (v/v) in water (A) and acetonitrile (B) at a flow of 0.4 mL/min. The gradient started from 98% A to 80% A in 10 min, followed by 80% A to 50% A in 10 min, followed by 50% A to 20% in 3 min, followed by 20% A to 0% in 5 min, and held for 2 min, then returned the initial gradient composition and allowed to equilibrate for 4 min.

Mass spectrometric analysis were collected in positive mode with full scan mode from 100 to 1200 *m*/*z*. The reference masses (121.0509 m/z for C_5_H_4_N_4_, and 922.0098 for C_18_H_18_O_6_N_3_P_3_F_24_) were continuously measured during the analysis to allow constant mass correction. The electrospray source was operated at 120°C; the cone gas and desolvation gas flow rates were set at 149 and 895 L/min, the capillary was 2.81 KV, respectively.

2. Result

2.1 Identification of compounds in PSM

Lots of compounds were displayed in the total ion chromatogram of water extract of PSM. Three compounds of paederosidic acid (1, 7.60 min), paederoside (2, 8.94 min), and paederosidic acid methyl ester (3, 10.46 min) were identified by comparing with the retention time and MS data of the reference compounds as depicted in Fig. S1.


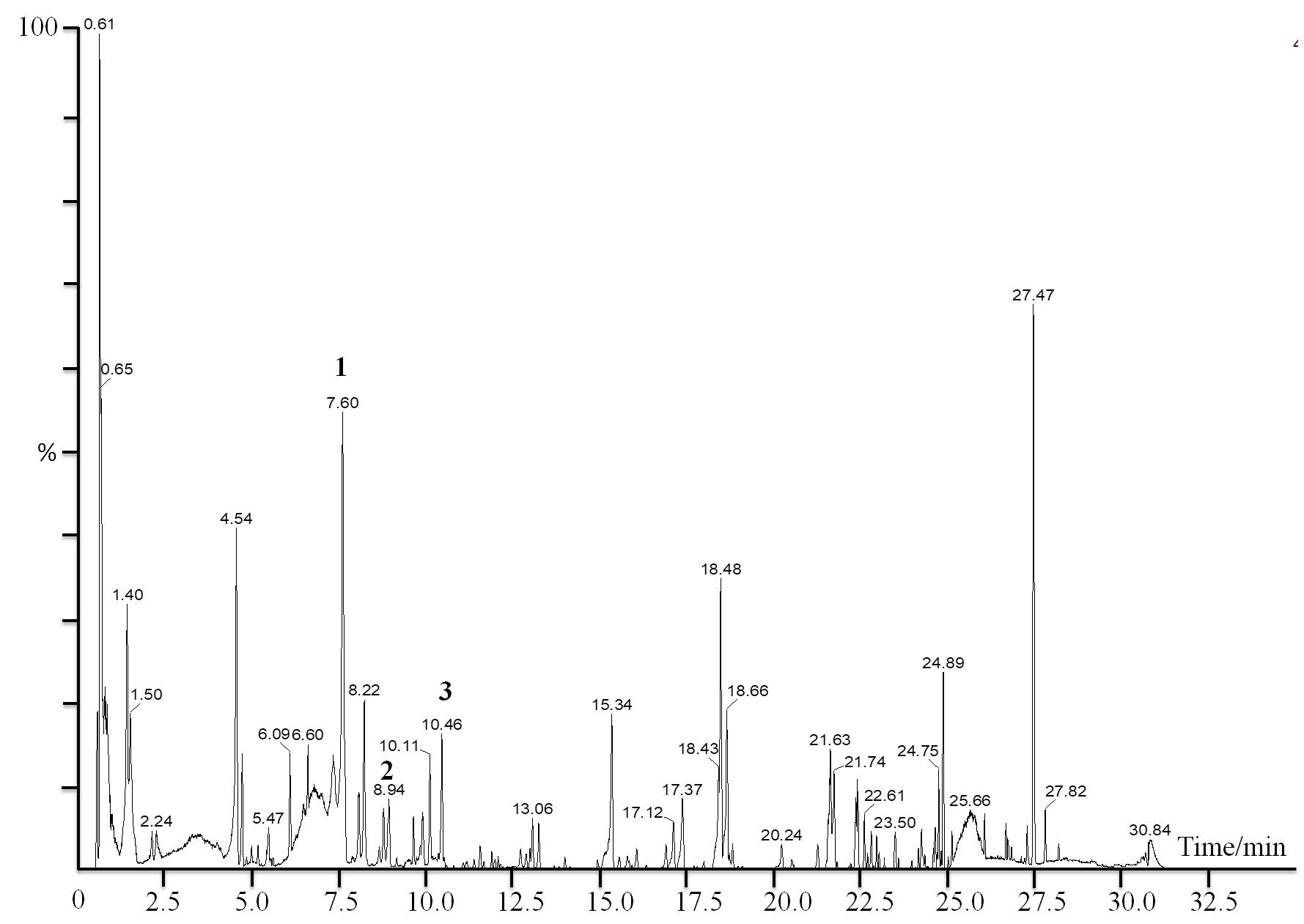
 Fig. S1. TIC of the water extract of PSM and the three identified compounds.


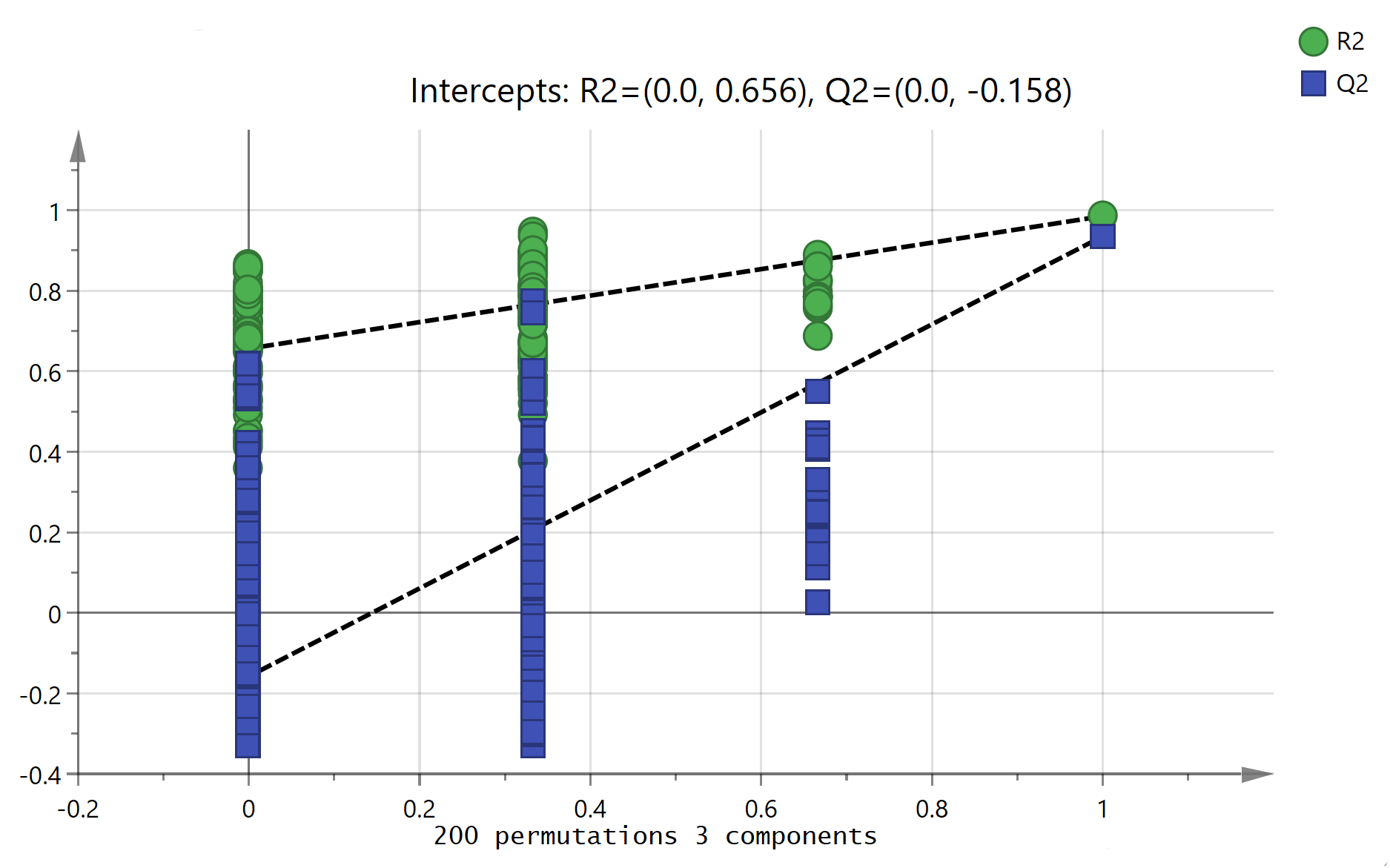


Fig. S2. The cross validation of the PLS-DA model (using 200 random permutations) to evaluate the validity of the mode.


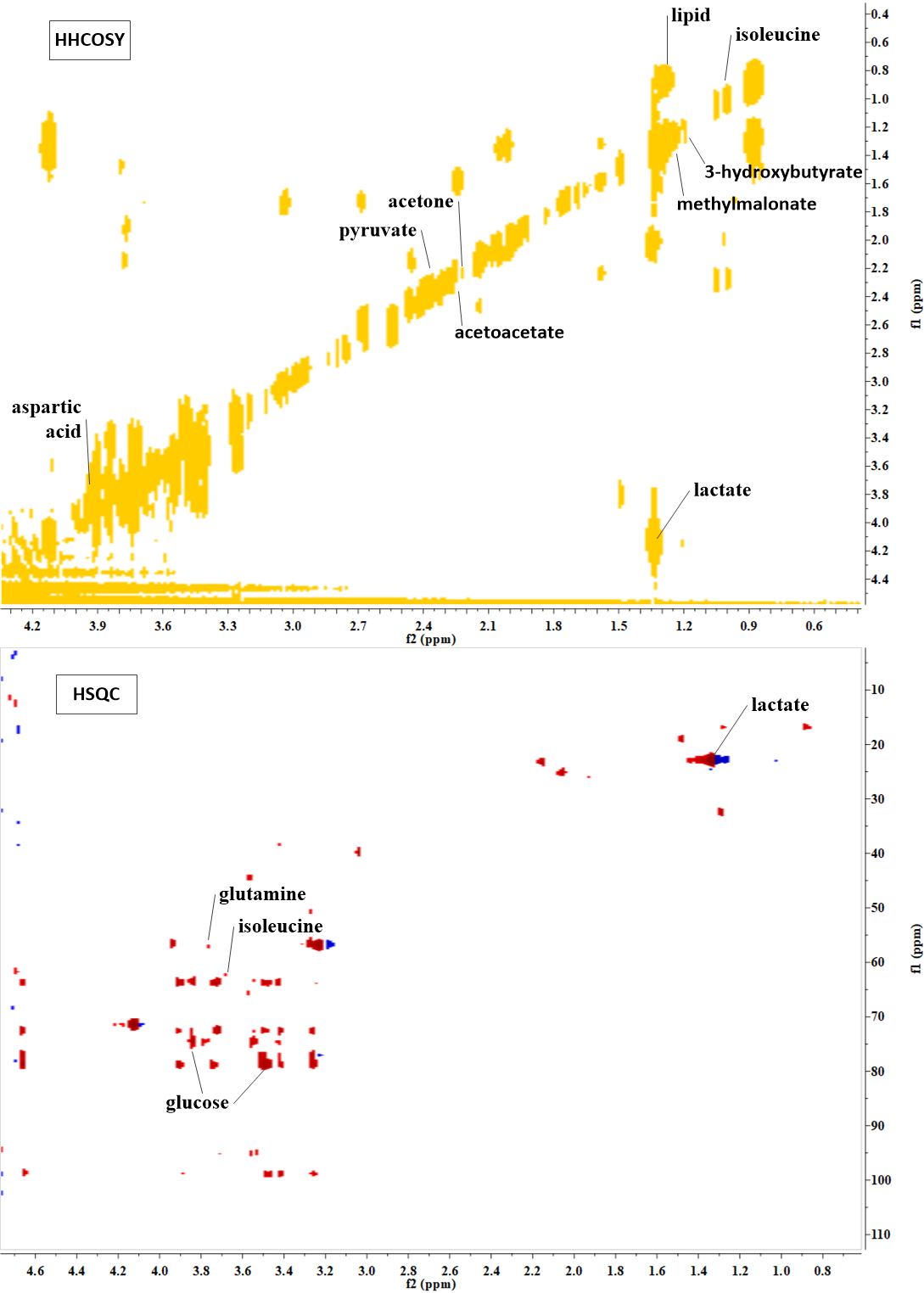


Fig. S3. ^1^H-^1^H COSY and HSQC of 2D ^1^H-NMR
